# Involvement of arginine 878 together with Ca^2+^ in mouse aminopeptidase A substrate specificity for N-terminal acidic amino-acid residues

**DOI:** 10.1371/journal.pone.0184237

**Published:** 2017-09-06

**Authors:** Pierre Couvineau, Hugo de Almeida, Bernard Maigret, Catherine Llorens-Cortes, Xavier Iturrioz

**Affiliations:** 1 Laboratory of central neuropeptides in the regulation of body fluid homeostasis and cardiovascular functions, INSERM U1050, Paris, France; 2 Center for Interdisciplinary Research in Biology (CIRB), Collège de France, Paris, France; 3 CNRS, UMR 7241, Paris, France; 4 Laboratoire Lorrain de Recherche en Informatique et ses applications (LORIA), Vandoeuvre Les Nancy, France; Russian Academy of Medical Sciences, RUSSIAN FEDERATION

## Abstract

Aminopeptidase A (APA) is a membrane-bound zinc metalloprotease cleaving, in the brain, the N-terminal aspartyl residue of angiotensin II to generate angiotensin III, which exerts a tonic stimulatory effect on the control of blood pressure in hypertensive animals. Using a refined APA structure derived from the human APA crystal structure, we docked the specific and selective APA inhibitor, EC33 in the presence of Ca^2+^. We report the presence in the S1 subsite of Arg-887 (Arg-878 in mouse APA), the guanidinium moiety of which established an interaction with the electronegative sulfonate group of EC33. Mutagenic replacement of Arg-878 with an alanine or a lysine residue decreased the affinity of the recombinant enzymes for the acidic substrate, α-L-glutamyl-β-naphthylamide, with a slight decrease in substrate hydrolysis velocity either with or without Ca^2+^. In the absence of Ca^2+^, the mutations modified the substrate specificity of APA for the acidic substrate, the mutated enzymes hydrolyzing more efficiently basic and neutral substrates, although the addition of Ca^2+^ partially restored the acidic substrate specificity. The analysis of the 3D models of the Arg-878 mutated APAs revealed a change in the volume of the S1 subsite, which may impair the binding and/or the optimal positioning of the substrate in the active site as well as its hydrolysis. These findings demonstrate the key role of Arg-878 together with Ca^2 +^ in APA substrate specificity for N-terminal acidic amino acid residues by ensuring the optimal positioning of acidic substrates during catalysis.

## Introduction

Aminopeptidase A (APA; EC 3.4.11.7) is a 160 kDa homodimeric type II Zn^2+^ membrane-bound aminopeptidase [1, 2]. APA cleaves the N-terminal glutamyl or aspartyl residue from peptide substrates such as angiotensin II (AngII) and cholecystokinin-8 and is activated by Ca^2+^ [3, 4]. Ca^2+^ not only enhances the hydrolysis by APA of N-terminal acidic amino acid residues from substrates, but also decreases the hydrolysis of N-terminal neutral or basic residues [5]. APA is expressed in various tissues, including the brush border of intestinal and renal epithelial cells and the vascular endothelium [6]. This enzyme is also expressed in several brain nuclei involved in the control of body fluid homeostasis and cardiovascular function, together with other components of the brain renin-angiotensin system [7]. Studies with the specific and selective APA inhibitor EC33 [(*S*)-3-amino-4-mercapto-butyl sulfonic acid] [8] have shown that, in the brain, APA generates angiotensin III (AngIII) [9] which exerts a tonic stimulatory action on the control of blood pressure in hypertensive animals and increases arginine vasopressin release [9–11]. Thus, the inhibition of brain APA with specific and selective inhibitors normalizes blood pressure in conscious hypertensive rats [10–13]. These studies suggest that brain APA constitutes a promising target for the treatment of hypertension [14–16]. This justifies the development of potent and selective APA inhibitors crossing the blood-brain barrier after oral administration for use as centrally-acting antihypertensive agents. With this aim, we have pursued our studies on the APA active site organization by molecular modeling and site-directed mutagenesis. First, using the crystal structure of leukotriene-A4 hydrolase (LTA4H; EC 3.3.2.6) [17] as a template and the functional information collected from previous site-directed mutagenesis studies on APA [18–24], we built a three-dimensional (3D) model of the mouse APA (mAPA) ectodomain from residue 79 to 559 [16, 24], including the catalytic domain. We subsequently docked into the APA active site, specific and selective APA inhibitors, such as GluPO_3_H_2_ (4-amino-4-phosphonobutyric acid) a transition state analog [25].

In the 3D model of APA, the Zn^2+^ atom is coordinated by the two histidine residues (His385 and His389) of the HEXXH motif, a water molecule, glutamate 408 (Glu408) of the WLNEG motif and either by one of the oxygen atoms of the phosphate of GluPO_3_H_2_ [24]. The water molecule bound to the Zn^2+^atom also interacts with Glu-352 and Glu-386 side chains [19, 21]. Ca^2+^ was then introduced in the APA 3D model. The Ca^2+^ atom was localized at the bottom of the S1 subsite, as suggested previously by Danielsen *et al*. [1]. The Ca^2+^ atom interacts with the acidic side chains of Asp-213 and Asp-218, the carbonyl group of Glu-215 and three water molecules, one of them being engaged in a hydrogen bond with the negatively charged carboxylate side chains of the inhibitors [26]. Site-directed mutagenesis of Asp-213 and Asp-218 (Asp-221 in human APA (hAPA)) showed that their negative charges are required for the presence of the Ca^2+^ atom that ensures acidic APA substrate specificity [26, 27]. More recently, the X-ray structure of human APA (residues 76 to 956) was resolved [28] and its comparison with the APA 3D model showed a perfect overlap of the catalytic regions.

In the present study, we have therefore taken advantage of the X-ray-derived structure of human APA to build a new system comprising the complete refined APA 3D structure complexed with EC33, an APA inhibitor exhibiting a inhibitory potency of 10^-7^M on APA and 100-fold less active on APN [8]. We visualized, in the S1 subsite, Arg-878 (Arg-887 in hAPA), which interacts with the electronegative sulfonate moiety of EC33 as observed by Yang *et al* in the crystal structure of human APA bound to a Glu, exhibiting an inhibitory potency of 7 x 10^−3^ M on APA [29]. The first data obtained by these authors on the role of Arg-887 in the absence of Ca^2+^ suggested an involvement in hAPA substrate specificity [28]. However, the Ca^2+^ increases APA preference for acidic substrates and this Ca^2+^-modulated APA substrate specificity is deemed physiologically relevant since the concentrations of Ca^2+^ that modulate APA activity, are in the same range as those found in brain fluid (i.e. 1–2 mM) [30]. Taking into account that Ca^2+^ plays a major role in APA substrate specificity, the aim of our work was to deepen the role of Arg-878 of mAPA together with Ca^2+^ in substrate/inhibitor binding and substrate specificity of APA for N-terminal acidic amino acid residues. For this purpose, we replaced Arg-878 with an alanine or a lysine residue and checked that the mutated enzymes displayed similar processing and subcellular distribution to wild-type mAPA. We then biochemically and kinetically characterized the purified recombinant wild-type and mutated enzymes with various substrates and determined their sensitivity to Ca^2+^ and various inhibitors exhibiting different side chains targeting the S1 subsite of APA.

## Materials and methods

### Materials

Restriction endonucleases Dpn1 was obtained from New England Biolabs Inc (Evry, France) and was used according to the manufacturer’s instructions. The PfuUltra High-fidelity DNA Polymerase was purchased from Agilent (les Ulis, France). The liposomal transfection reagent, Lipofectamine 2000, the pcDNA 3.1-His vector and the monoclonal anti-Xpress antibody were purchased from Life Technologies (Cergy-Pontoise, France). *CHO-K1* cells were obtained from American Type Culture Collection (Manassas, VA, USA). The horseradish peroxidase-conjugated sheep anti-mouse antibody was purchased from Sigma-Aldrich (Saint Quentin Fallavier, France). The cOmplete, EDTA-free Protease Inhibitor Cocktail was purchased from Roche (Mannheim, Germany). Immobilized cobalt affinity columns (Talon) were obtained from Clontech (Heidelberg, Germany). The synthetic substrates, GluNA, AspNA, AlaNA and LysNA were purchased from Bachem (Bunderdorf, Switzerland).

### Molecular docking and molecular dynamics simulations

The Crystallographic structure of human aminopeptidase A complexed with glutamate and calcium (PDB ID: 4KXD) was used to perform docking and molecular dynamics simulations. The 3D-structure was first treated to remove the Glu ligand, as well as the N-Acetyl-D-Glucosamine chains and non-conserved water molecules. Zn^2+^ and Ca^2+^ atoms were kept, and a loop was added for residues missing from crystallographic data (residues 608 to 611) using the software MODELLER [31]. Reference inhibitors developed by our group were docked into our 3D-model using GOLD v5.1 [32], with the ChemPLP scoring function selected and several penalties added based on the analysis of available crystallographic structures. The list of docked molecules and the applied penalties can be found in S3 and S4 Tables, respectively. In order to analyze if the predicted interactions would be conserved in a physiological and dynamical molecular system, compounds EC33 and GluPO_3_H_2_ were chosen to be parametrized, and poses obtained from the automated docking process above were submitted to molecular dynamics (MD) simulations using NAMD. Briefly, small ligands were parametrized with Discovery Studio Visualizer 4.5 (BIOVIA) using CHARMm 36 forcefields [33, 34], with atom types being assigned manually. Ligand-protein complexes were generated with VMD autopsf plug-in using the BIOVIA provided topology files. The MUTATOR plugin was used to build systems with mutated hAPAs (R887A and R887K). The obtained systems were then included in a water-box using the VMD solvate plug-in, followed by charge neutralization through the ionize plug-in. The final systems comprising ~80,000 atoms were then subjected to three stages of conjugate gradient energy minimization of 6400 steps each. The first stage had the ligand-protein complex fixed, and allowed only water to move freely. The second stage allowed ligand motions, and the last stage consisted of the energy minimization of the entire system. Those systems were then subjected to a short MD simulation of 500 ps in order to equilibrate pressure and the temperatures before proceeding to production runs. Each MD production run simulation was carried out in the NPT ensemble, with periodic boundary conditions, Particle Mesh Ewald for treating electrostatic interactions and 1 fs per time step for 50,000,000 steps, generating a trajectory of 50 ns. Trajectory analyses were performed with VMD, pair interaction calculations with NAMD and ligand binding site volume with MDPOCKET.

### Cloning and site-directed mutagenesis

The cDNA encoding mouse APA [22] was used as a template for the generation of mutants by PCR-based site-directed mutagenesis. The arginine residue at position 878 was replaced with either an alanine or a lysine residue. The mutated primers A (forward) and B (reverse) were:

A (5’-CAATCAATGACGCATACCTTGGCCGGATCG-3’) and B (5’-GGCCAAGGTATGCGTCATTGATTGTAAATC-3’) for R878A

A (5’-CAATCAATGACAAATACCTTGGCCGGATCG-3’) and B (5’-GGCCAAGGTATTTGTCATTGATTGTAAATC-3’) for R878K

The underlined bases encode the new amino acid residue and replace the naturally occurring codon at position 2634. Nucleotide numbering corresponds to the mAPA sequence deposited in the GenBank/EBI database (accession number M29961). PCR was performed with PfuUltra High-fidelity DNA Polymerase to make a full-length plasmid PCR product. The methylated plasmid used as the template was then specifically digested with Dpn1 and Top 10 *E*. *coli* were transformed to obtain the mutated cDNA. Sequences of all constructs were checked by DNA Sequencing (GATC Biotech, Mulhouse, France).

### Cell culture and establishment of stable CHO-K1 cell lines producing wild-type and mutated His-mAPAs

Chinese Hamster Ovarian (CHO—American Type Culture Collection) cells were cultured in Ham’s F-12 medium supplemented with 10% fetal calf serum, 0.5 mM glutamine, 100 units/ml penicillin and 100 μg/ml streptomycin (all from Life Technologies) at 37°C in a humidified atmosphere of 5% CO_2_, 95% air. The cells were transfected with either 2.5 μg of plasmid containing the Xpress-polyHis-tagged wild-type or the Xpress-polyHis-tagged mutated mAPA cDNAs using Lipofectamine 2000 following manufacturer instructions. Stable cell lines producing the wild-type and mutated His-APAs were established as previously described [23].

### Immunofluorescence analysis of stably transfected CHO cells

Stable transfected CHO cells producing wild-type and mutated His-mAPAs were seeded (100,000 cells) onto 14-mm-diameter coverslips and grown for 24h. Then, the cells were fixed and permeabilized in 100% ice-cold methanol for 5 min on ice. After blocking with 0.5% BSA in 0.1 M PBS, coverslips were incubated with the anti-Xpress antibody (1:1000) in 0.5% BSA in PBS for 2h at 20°C, washed thrice with cold PBS and then incubated with a 1:500 dilution of AlexaFluor 488-conjugated polyclonal anti-rabbit antibody in 0.5% BSA in PBS for 2 h at 20°C. The coverslips were then mounted in Mowiol. The cells were examined with a Leica TCS SP V (Leica Microsystem, Heidelberg, Germany) confocal laser scanning microscope equipped with an argon/krypton laser and configured with a Leica DM IRBE inverted microscope. AlexaFluor was detected after 30% excitation at 488 nm. The images (1024 x1024 pixels) were obtained with a 63x magnification oil-immersion objective. Each image corresponded to a cross-section of the cells.

### Purification and western blot analysis of wild-type and mutated recombinant His-mAPAs

Stable transfected CHO cells (3x10^9^ cells) were harvested and a crude membrane preparation was obtained, as previously described [23]. Crude membrane preparation was solubilized overnight in 50 ml of 20 mM Tris-HCL buffer (pH 8) containing 100 mM NaCl and 1% nonidet P-40 (loading buffer, 1 ml/10^6^ cells ratio). The solubilized membrane preparation was centrifuged at 100,000 x g for 90 min and the supernatant was subjected to metal affinity chromatography with a metal chelate resin column (Talon Co^2+^, Clontech). The wild-type or mutated His-mAPAs were eluted with loading buffer containing 250 mM imidazole. The eluted fractions were loaded into Pierce Protein Concentrators, 150K MWCO (ThermoFisher) to concentrate the fraction and to replace the elution buffer with 20 mM Tris-HCl buffer (pH 7.4) containing 100 mM NaCl and 0.1% nonidet P-40. Protein concentrations were determined with the BCA assay kit (Calbiochem). The purity of the final preparation was assessed by NuPAGE (4–12% polyacrylamide gels, Invitrogen) and SilverQuest Silver staining (all from Life Technologies). Purified wild-type and mutated His-mAPAs were resolved by 4–12% SDS-PAGE and the proteins were transferred on a nitrocellulose membrane. His-tagged recombinant proteins were detected with the anti-Xpress antibody (1:5000 dilution). The resulting immune complex was detected with an anti-mouse antibody coupled to HRP (GE Healthcare, 1:10,000) and revealed with the ECL Plus Western blotting detection system (GE Healthcare) and image was acquired with an ImageQuant LAS 4000 (GE Healthcare).

### Enzyme assay

Enzyme assays were carried out for wild-type and mutated His-mAPAs in 50 mM Tris-HCl buffer, pH 7.4, with or without 4 mM CaCl_2_, by monitoring the rate of hydrolysis of GluNA, as previously described [19]. The sensitivity to Ca^2+^ of purified wild-type and mutated His-mAPA was determinated by incubating 0.5 mM GluNA with 0 to 4 mM CaCl_2_ in a final volume of 100 μl of 50 mM Tris-HCl buffer, pH 7.4. All the assays were performed in black 96-well plates (solid black 96-well flat-bottomed non-treated plates with no lid; Corning Costar). All of the enzymatic studies were performed such that substrate hydrolysis rates were maintained below 10% (initial rate conditions). The kinetic curves were monitored by following the increase in fluorescence at 460 nm (*l*_ex_ = 330 nm), induced by the release of the β-naphthylamide fluorogenic part of the substrate by APA. Fluorescence signals were monitored by counting photons with a spectrophotometer (Fusion universal microplate analyser; Packard) equipped with a temperature control device and a plate shaker. The kinetic parameters (*K*_*m*_ and *k*_*cat*_) were determined from Michaelis-Menten plots, using GraphPad Prism 4 software (GraphPad) with a final concentration of substrate (GluNA) from 0.005 to 5 mM with or without 4 mM Ca^2+^. For evaluation of the substrate specificity of APA with or without 4 mM Ca^2+^, the rate of hydrolysis of two additional substrates, α-L-aspartate-β-naphthylamide (AspNA), α-L-alanine-β-naphthylamide (AlaNA) and basic α-L-lysine-β-naphthylamide (LysNA), was determined under the same experimental conditions as described for GluNA. The sensitivity of wild-type and mutated His-mAPAs to inhibition by [(3S)-3-amino-4-sulfanyl-butane-1-sulfonic acid] (EC33), glutamate-thiol (GluSH), glutamate-phosphonic-acid (GluPO_3_H_2_), methionine-thiol (MetSH) and lysine-thiol (LysSH) was determined by establishing dose-dependent inhibition curves for a final GluNA concentration of 0.5 mM in the presence or absence of 4 mM CaCl_2_. These compounds being linear competitive inhibitors, their *K*_*i*_ were calculated from the formula *K*_*i*_ = IC_50_/ (1+ [GluNA]/*K*_*m*_).

### Statistical analysis

Statistical comparisons were carried out with Student's unpaired t-test. Differences were considered significant if *p* was less than 0.05.

## Results and discussion

APA belongs to the monozinc aminopeptidase family, which requires Zn^2+^ for catalytic activity. In addition, the substrate specificity of APA, unlike that of other monozinc aminopeptidases, is enhanced by Ca^2+^. Indeed, APA preferentially hydrolyzes peptides with N-terminal acidic amino acids and this is due in part to the presence of Ca^2+^ in the S1 subsite of the APA active site [22, 35]. In the present work, we used the recent crystal structure of human APA to build a 3D model of APA complexed with EC33, a specific and selective APA inhibitor targeting the S1 subsite to explore if other residues, in addition to Ca^2+^, participate to APA acidic substrate specificity.

### Molecular modeling of human APA and docking of the APA inhibitor EC33

**Fig 1:**
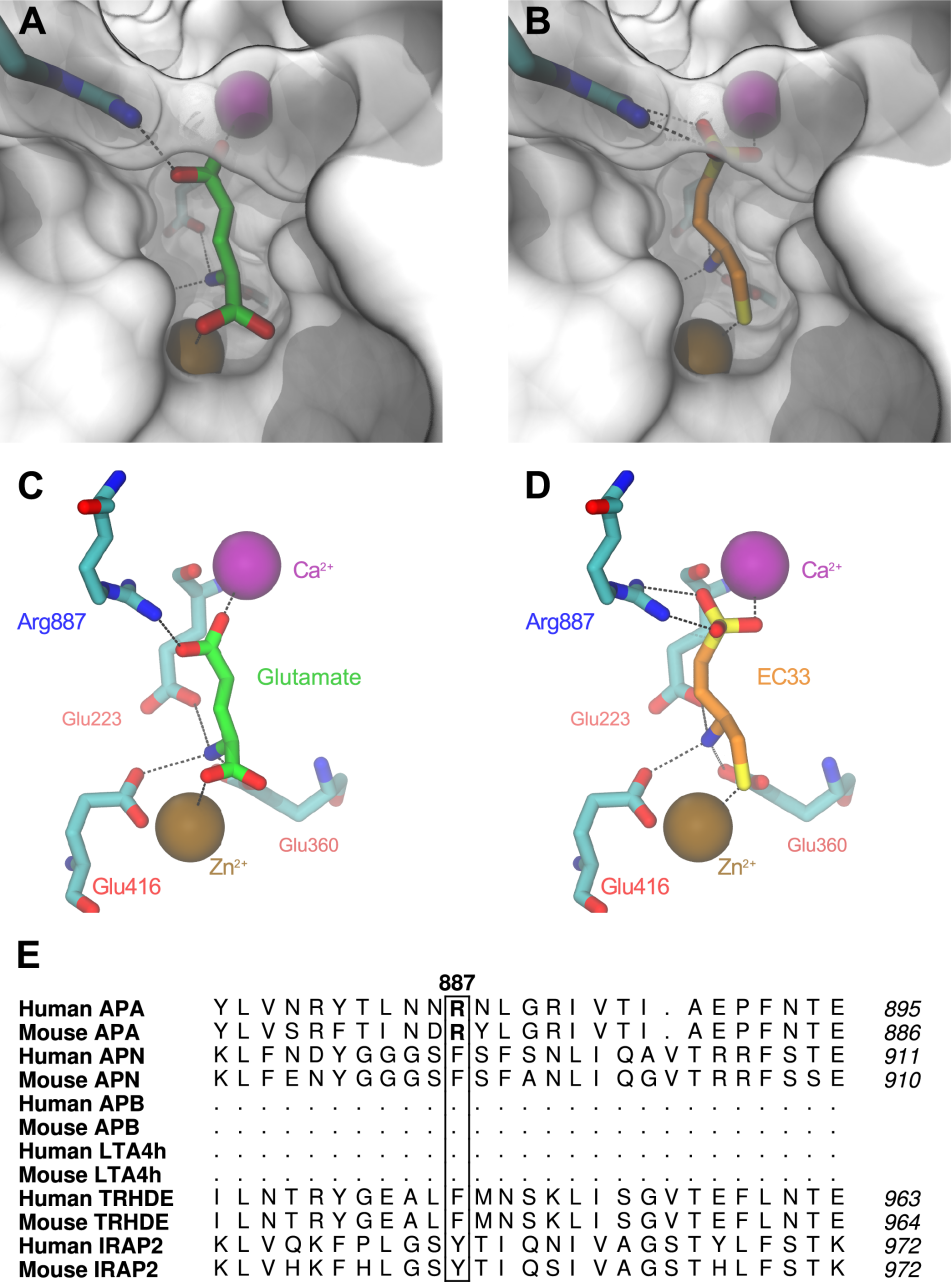
Human Aminopeptidase A with bound glutamate in the crystallographic structure and the proposed binding mode of the inhibitor EC33 after molecular docking in the 3D-model of human APA. (A and C), S1 subsite visualized in the crystallographic structure of APA co-crystallized with the glutamate (green) (PDB ID 4KXD). **(**B and D), S1 subsite visualized in the 3D-model of human APA after molecular docking of EC33 (orange). Protein residues either blocking the visualization or interacting with ligands are represented with a transparent surface. Predicted hydrogen or metallic bonds are drawn as dashed lines. Interacting protein residues are labeled (C and D) accordingly to the human APA numeration. (E), Alignment of the human and mouse APA amino acid sequences with the sequences of other monozinc aminopeptidases from the M1 family. Residues in bold are the Arg-887 of human APA and the Arg-878 of mouse APA. APA, Aminopeptidase A; APN, Aminopeptidase N; APB, Aminopeptidase B; LTA4H, Leukotriene A4 hydrolase; TRHDE, Thyrotropin releasing degrading enzyme; IRAP, Insulin regulated aminopeptidase.

Using the recent crystal structure of hAPA (PDB ID 4KXD, [28]), with a bound Glu (Fig 1A and 1C), we built a 3D model of hAPA complexed with EC33 (Fig 1B and 1D). EC33 interacted in the S1 subsite of the APA active site with the Zn^2+^ atom via its thiol group and Glu-215, Glu-352 and Glu-408 (Glu-223, Glu-360 and Glu-416 in hAPA) via their free alpha amino group. [24]. In addition, the sulfonate group of EC33 was embedded at the bottom of the S1 subsite formed by Asp 221 and Asp 226, which bind the Ca^2+^ atom. All these interactions were previously visualized in the 3D model of the mouse APA ectodomain from residues 79 to 559 that we generated by homology using the X-ray crystal structure of leukotriene A4 hydrolase (LTA4H; EC 3.3.2.6) as a template and the functional data collected from site-directed mutagenesis studies on APA [17].

The crystal structure of APA with a bound Glu showed the presence of Arg-878 (Arg-887 in hAPA) at the bottom of the S1 subsite, interacting with the electronegative carboxylate of Glu. We also visualized the presence of this residue in the S1 subsite of the 3D model of human APA/EC33 complex, the guanidinium moiety of which established an interaction with the sulfonate groups of EC33. Moreover, multiple sequence alignments of monozinc aminopeptidases (Fig 1E) demonstrated the presence of this Arg residue exclusively in APA.

Yang *et al* substituted this residue by an Ala and showed a decrease in GluNA hydrolysis and an increase in ArgNA hydrolysis by the mutated APA versus wild-type APA, all these experiments being performed in the absence of Ca^2+^ [28], suggesting that Arg-878 could participate to APA substrate specificity. However, when conducting these experiments in the absence of Ca^2+^ might influence the results since one of the key features of APA is its stimulation by Ca^2+^ [1]. The enzymatic activity of APA with substrates bearing acidic amino acids at their N-terminal part increases 5 to 7-fold when the Ca^2+^ concentration rises from 0 to 4 mM [2, 22]. Ca^2+^ not only enhances the hydrolysis by APA of N-terminal acidic residues from substrates, but also reduces the hydrolysis of neutral or basic residues [5]. Furthermore, we have shown, by molecular modeling and site-directed mutagenesis studies, that Asp-218 and Asp-213 bind the Ca^2+^ atom into the S1 subsite [26] and that Ca^2+^ interacts with the side chain carboxylate of the P1 residue, ensuring the substrate specificity of APA for N-terminal acidic amino acid residues. The high degree of sequence identity (77%) between hAPA and mAPA (S1 Fig) suggested that both proteins have closely related structures. We therefore investigated the role of Arg-878 of mAPA together with Ca^2+^ by molecular modeling and site-directed mutagenesis.

### Site-directed mutagenesis, expression and purification of recombinant wild-type and mutated His-mAPAs

**Fig 2:**
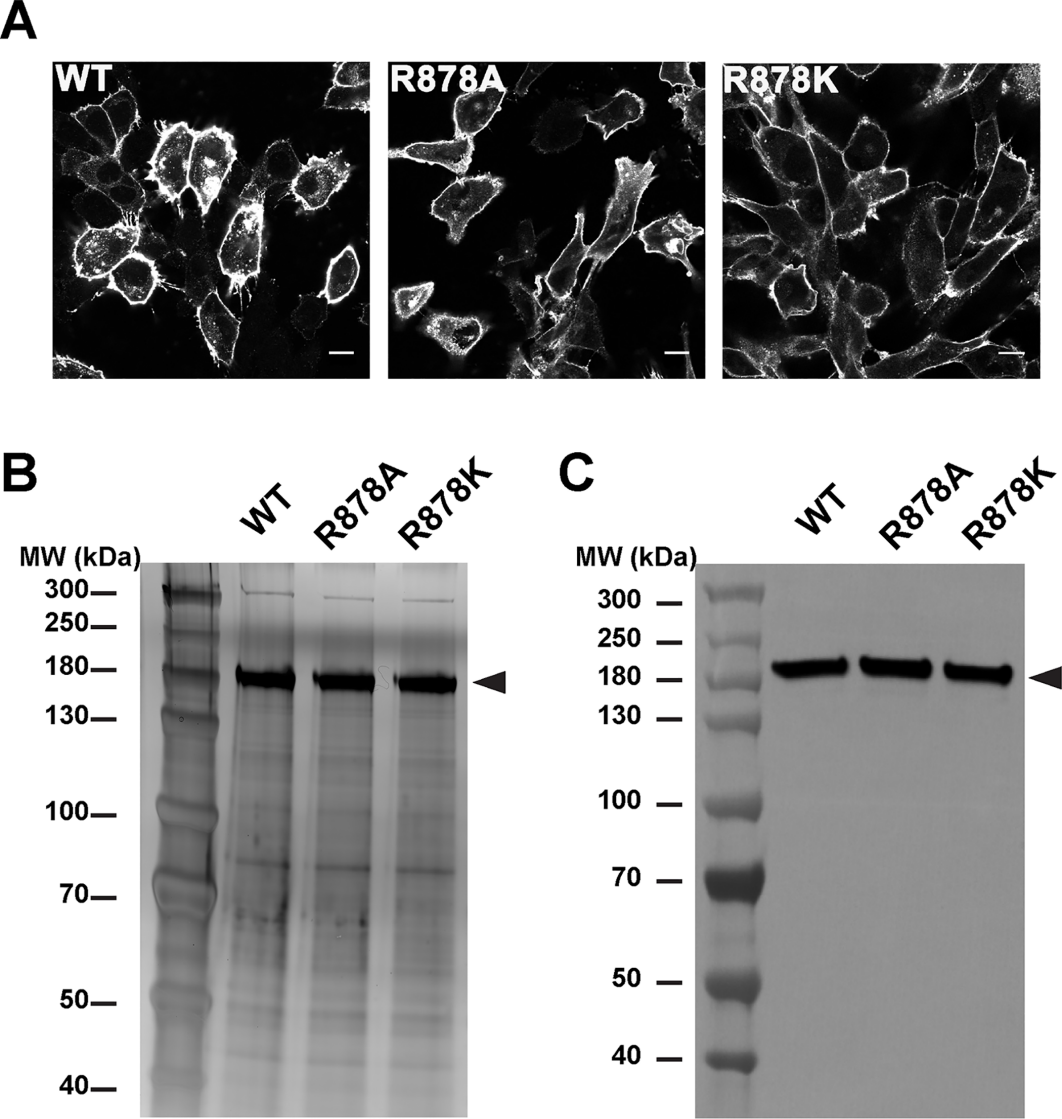
Expression and purification of wild-type and mutated mouse recombinant His-APAs. **(**A), Transfected CHO cells stably expressing the wild-type or mutated Xpress-His-mAPA construct were fixed and immunolabeled with a mouse monoclonal anti-Xpress primary antibody, which was detected with an Alexa 488-conjugated anti-mouse secondary antibody. Immunofluorescence was monitored by confocal microscopy. The *bar* indicates 10 μm. (B and C), Purified recombinant wild-type and mutated Xpress-His-mAPA were analyzed by SDS-PAGE 4–12% and proteins were silver stained (B) or transferred into nitrocellulose membrane and subjected to Western blot analysis using mouse monoclonal anti-X-press antibody (C). The arrows indicate the monomeric form of APA.

For this purpose, we replaced Arg-878 with an alanine (R878A) or a lysine (R878K) residue by site-directed mutagenesis. Wild-type and mutated His-mAPAs were then stably expressed in CHO cells. We first verified that the mutations did not affect the production and the processing of the recombinant proteins by investigating the subcellular distribution of wild-type and mutated His–mAPAs in stably transfected CHO cells by immunofluorescence staining. Confocal microscopy analysis of wild-type and mutated His-mAPAs showed that all recombinant His-mAPAs were located at the plasma membrane consistent with the correct processing of these enzymes (Fig 2A). Wild-type and mutated His-mAPAs stably expressed in CHO cells were then purified by metal affinity chromatography and purified proteins were subjected to SDS-PAGE/silver staining analysis (Fig 2B) and Western blot analysis (Fig 2C). We showed that all recombinant His-mAPAs displayed a major mature form of 180-kDa (Fig 2B and 2C) that corresponded to the mature monomeric glycosylated APA sorted from the Golgi apparatus. SDS-PAGE and silver staining analysis showed that the enzymes reached a purity higher than 90% with an overall yield of 72% and a purification factor of 118- fold.

### Enzymatic activity of purified wild-type and mutated His-mAPAs

**Fig 3:**
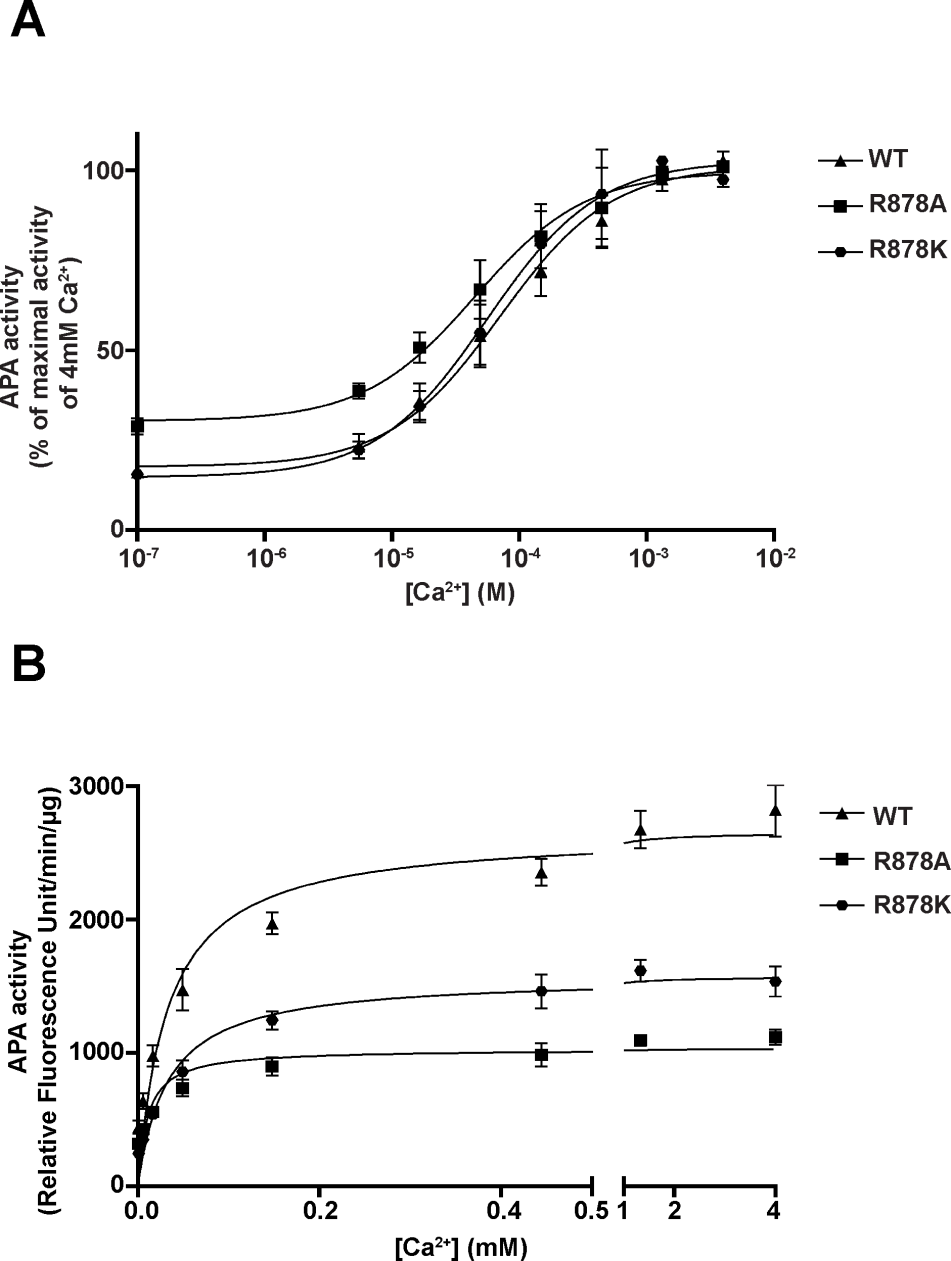
Effects of the divalent cation Ca^2+^ on recombinant wild-type and mutated mouse APAs enzymatic activities. Purified mAPAs (2 μg) were incubated at 37°C for 30 min with 0.5 mM GluNA with various concentrations of CaCl_2_ in a final volume of 100 μl of 50 mM Tris-HCl buffer (pH7.4). (A), APA enzymatic activities expressed as a percentage of maximal hydrolysis velocity obtained with 4 mM Ca^2+^ for each purification *vs* log of Ca^2+^ concentration. (B), Effect of increasing Ca^2+^ concentration on APA enzymatic activities.

We then determined the Ca^2+^ activation profile of purified recombinant wild-type and mutated His-mAPAs, using an acidic substrate, α-L-glutamyl-β-naphthylamide (GluNA) as previously described [22]. We observed that wild-type mAPA was strongly activated by Ca^2+^ (EC_50_ = 22 ± 5 μM), with enzymatic activity increasing by 7-fold as the Ca^2+^ concentration raised from 0 to 4 mM, as previously reported [22]. The replacement of Arg-878 by a lysine residue, conserving the positive charge, did not change the ability of this enzyme to be activated by Ca^2+^ when compared to that of the wild-type. By contrast, the replacement of Arg-878 by an alanine, a neutral residue devoid of charge, very slightly reduced activation by Ca^2+^ (Fig 3A). This finding is consistent with the localization of Arg-878 (Arg-887 in hAPA) into the APA active site 3D model, but showed that Arg-878 does not contribute to Ca^2+^ binding in the S1 subsite. However, the maximal enzymatic activities of R878A and R878K mutated mAPAs measured in the presence of a supramaximal concentration of Ca^2+^ were significantly decreased (by a factor of 2.4 and 1.7, respectively, (Fig 3B) when compared to wild-type mAPA. Since these mutated enzymes exhibited a similar pattern of membrane expression when compared to wild-type mAPA, these defect suggest that Arg-878 could contribute to substrate recognition and hydrolysis.

### Kinetic parameters for purified recombinant wild-type and mutated His-mAPAs

This led us to determine the kinetic parameters (*K*_*m*_ and *k*_*cat*_) for wild-type and mutated His-APAs in the absence or presence of 4 mM Ca^2+^ using GluNA as a substrate. The results are summarized in Table 1. In the absence of Ca^2+^, the cleavage efficiency (*k*_*cat*_*/K*_*m*_*)* of the mutated enzymes for the acidic substrate, GluNA was clearly reduced when compared to that of the wild-type, but less markedly (by a factor 25) for R878A than for R878K (by a factor 281). This was due to a net decrease in the affinity of the mutated enzymes for GluNA together with a slight decrease in hydrolysis velocity. The addition of Ca^2+^ enhanced wild-type APA activity by increasing the affinity of the enzyme for GluNA as well as substrate hydrolysis velocity, increasing cleavage efficiency (*k*_*cat*_*/K*_*m*_*)* by 25-fold. In the same conditions, a lower cleavage efficiency of the mutated enzymes for GluNA was observed when compared to wild-type but less markedly than in the absence of Ca^2+^, by a factor 21 for R878A and a factor 4 for R878K. This was due to a decrease in the affinity of both mutated enzymes for GluNA (by a factor 6) but also to a decrease in hydrolysis velocity for R878A (by a factor 4) whereas the *k*_*cat*_ for R878K was unchanged. These findings suggested that Arg-878 of mAPA participates in the establishment of the Michaelis complex as well as in catalysis either in the presence or in the absence of Ca^2+^.

**Table 1:** Kinetic parameters for wild type and mutated mAPAs in the absence or presence of Ca^2+^ using HGluβNA as a substrate.

Recombinant APAs	*No Ca*^*2+*^			*4 mM Ca*^*2+*^		
	*K*_*m*_ (μM)	*k*_*cat*_ (s-1)	*k*_*cat*_/*K*_*m*_ (s-1/mM)	*K*_*m*_ (μM)	*k*_*cat*_ (s-1)	*k*_*cat*_/*K*_*m*_ (s-1/mM)
Wild type	165 ± 13.5	13.9 ± 0.1	84.3	39.1 ± 3.9	81.7 ± 4.0	2090
R878A	1500 ± 79.6***	4.95 ± 0.1***	3.3	221 ± 4.0***	22.4 ± 0.1***	102
R878K	12500 ± 1450***	3.12 ± 0.4***	0.3	269 ± 5.1***	135 ± 1.3***	502

*K*_*m*_ and *k*_*cat*_ values are the mean ± S.E.M from six to eight separate experiments performed in duplicate

*** *p*<0.001, significant when compared to the corresponding wild type value

### Inhibitory potency of various classes of compounds against GluNA on purified recombinant wild-type and mutated His-mAPAs

To further investigate the functional role of Arg-878, we evaluated the binding mode of various inhibitors mimicking the Michaelis complex such as the β-amino thiol inhibitors, EC33, GluSH, LysSH and MetSH by measuring their inhibitory potencies (*K*_*i*_) against GluNA hydrolysis by wild-type and mutated His-mAPAs in the absence or in the presence of 4 mM Ca^2+^. These compounds interact with the amine binding site, within the S1 subsite via their acidic, basic, or neutral side chains, and with the Zn^2+^ atom via their chelating thiol group. We also used the inhibitor, GluPO_3_H_2_ which behaves as an analog of the transition state, mimicking the interaction of the substrate with the enzyme at the transition state by its phosphonate group that mimics the tetrahedral intermediate formed during catalysis. The structures of the inhibitors and the results are summarized in Table 2 and Table 3.

**Table 2:** *Ki* values (nM) for several inhibitors with wild-type and mutated recombinant mAPAs targeting the S1 subsite in absence of calcium.

	WT	R878A	R878K
**GluSH**	1570 ± 270	1720 ± 190^n.s^	> 10000
**MetSH**	750 ± 46	475 ± 140***	1250 ± 57***
**LysSH**	317 ± 30	118 ± 13***	378 ± 20^n.s^

*Ki* values are the mean ± S.E.M from seven separate experiments with duplicate determinations

*** *p*<0.001 and n.s (not significant) when compared with the corresponding wild type values

**Table 3:** *Ki* values (nM) of several inhibitors with wild-type and mutated mAPAs targeting the S1 subsite in presence of calcium.

	WT	R878A	R878K
**GluSH**	314 ± 33	983 ± 160***	2100 ± 440***
**EC33**	268 ± 22	2000 ± 200***	1520 ± 126***
**GluPO**_**3**_**H**_**2**_	74 ± 7.7	6500 ± 240***	619 ± 21***
**MetSH**	9180 ± 770	3220 ± 200***	5140 ± 560***
**LysSH**	13400 ± 1700	7510 ± 660***	13900 ± 390^n.s^

*Ki* values are the mean ± S.E.M from seven separate experiments with duplicate determinations

*** *p*<0.001 and n.s (not significant) when compared with the corresponding wild-type values

In the absence of Ca^2+^, we showed that R878A was slightly better inhibited (maximally inhibited by a factor of 2) by basic (LysSH) or neutral (MetSH) inhibitors than wild-type APA whereas R878K was less inhibited by acidic (GluSH) or neutral inhibitors than wild-type. The presence of 4 mM Ca^2+^ enhanced GluSH inhibitory potency on wild-type mAPA, R878A and R878K mutants by 5.0, 1.7 and 6.6-fold, respectively, when compared to the corresponding *K*_i_ values obtained in the absence of Ca^2+^. In contrast, the presence of Ca^2+^ decreased the inhibitory potency of MetSH and LysSH on wild-type and mutated APAs by a factor of 12 and 42 for wild-type, 7 and 64 for R878A and 4 and 37 for R878K when compared to the *K*_i_ values obtained in the absence of Ca^2+^. This showed that the presence of Ca^2+^ partially restored inhibitor specificity of the mutated mAPAs for inhibitors with a N-terminal acidic amino acid residue (GluSH>MetSH>LysSH), demonstrating that the Ca^2+^ atom is a major component of APA acidic inhibitor specificity. However, the affinity of EC33 and GluSH for the mutated enzymes (R878A and R878K) remains weaker (factor 3 to 9) than that for the wild-type suggesting that the interaction between the acidic side-chain of the inhibitors with APA required the positive charge of Ca^2+^ in the S1 subsite but not only. Indeed, our data showed that the conservative charge of the lysine substitution is not enough to maintain an optimal positioning of the acidic inhibitors into the active site versus wild-type mAPA. This can be explained by a different orientation of the inhibitor due to the longer side chain of the lysine residue when compared to that of the arginine. Since our data showed that Arg-878 plays a role not only in the establishment of the Michaelis complex but also in catalysis, we investigated the effects of the mutations of Arg-878 on GluPO_3_H_2_ binding. GluPO_3_H_2,_ in the presence of Ca^2+^, inhibited R878A 87-times less efficiently than wild-type, whereas it inhibited R878K 8-times less efficiently than wild-type. This different behavior of GluPO_3_H_2_ regarding the mutated enzymes is in agreement with data obtained on the hydrolysis of GluNA. Altogether, our data suggested that Arg-878 played a key role in substrate specificity of mAPA towards substrates with a N-terminal acidic amino acid residue by contributing with the Ca^2+^ in the S1 subsite to interactions with the electronegative charge of the P1 substrate side chain.

### Molecular dynamics simulation analysis

**Fig 4:**
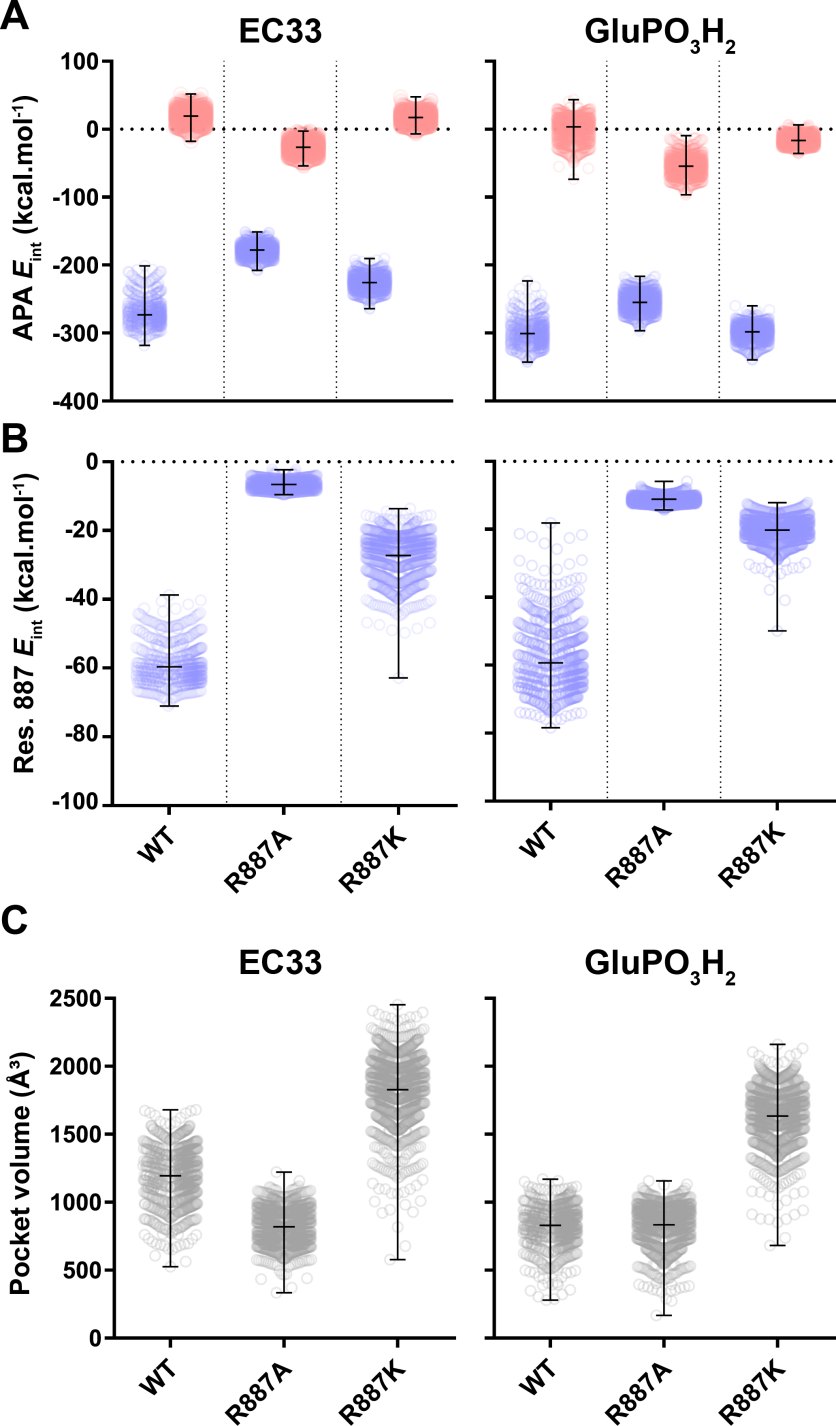
Analysis of the molecular dynamics simulations trajectories reveals loss of inhibitor affinity for the mutated hAPAs and a larger active site pocket for the R887K mutant. (A), The pair interaction energies analysis between inhibitor and protein (blue) or inhibitor and solvent (red) indicate less spontaneous interactions between inhibitors and the mutant R887A when compared to wild-type hAPA. (B) Pair interaction energies analysis between residue 887 in the wild-type or mutated hAPAs and the inhibitors shows that the Arg-887 from the wild-type APA and the Lys-887 of the R887K mutant spontaneously interact with EC33 and GluPO_3_H_2_. (C), Pocket volume was extracted from the trajectories after removing the docked inhibitors, showing that the R887K mutant exhibits a larger binding pocket. Each point in the scatter plots correspond to the values calculated from a different frame in the MD trajectories, vertical bars illustrate the minimum and maximum values for each calculation, horizontal bars correspond to the means.

To better understand the effects of mutations on inhibitor binding and substrate accessibility, we performed MD simulations with wild-type and mutated hAPAs in the presence of Ca^2+^ and various inhibitors. Pair interaction analysis revealed that the docked inhibitors (EC33 and GluPO_3_H_2_) were kept stable at the active site of wild-type hAPA during the entire simulation (Fig 4A) and the average interaction energies between the inhibitor and the protein versus those obtained between the inhibitor and the solvent were determined (Fig 4A). Wild-type displayed an average interaction energy of -270.1 ± 17.5 kcal/mol for EC33, and -299.5 ± 17.6 kcal/mol for GluPO_3_H_2_. Following virtual mutation, those energies became less spontaneous (less negative), but still favorable for inhibitor binding, agreeing with our experimental data. The substitution of Arg-887 by an alanine (R878A mutant in mAPA) decreased the binding of the inhibitors with an average interaction energy of -278.1 ± 8.9 kcal/mol for EC33 and of -254.5 ± 13.3 kcal/mol for GluPO_3_H_2_. Regarding the more conservative R887K mutation, interaction energies were close to those of the wild-type model (-225.6 ± 10.9 kcal/mol for EC33 and -298.9 ± 11.6 kcal/mol for GluPO_3_H_2_). Indeed, the substitution of Arg-887 by an alanine leads to a strong decrease in the energy of interaction of GluPO_3_H_2_ with the hAPA active site that can be due to a major modification of the electropositive environment of the S1 subsite. In addition, the higher interaction between GluPO_3_H_2_ and the solvent observed with the R887A mutant led to a decrease in the affinity of the inhibitor towards the R887A mutated enzyme. On the other hand, the fact that the inhibitory potency of GluPO_3_H_2_ towards the R878K mutant is less impacted than that towards R878A mutant, could be explained by the fact that the ε amine moiety of the lysine residue maintains the electropositive environment of the S1 subsite. This hypothesis is further supported by the fact that isolated pair interaction analysis performed between single residues and the inhibitors revealed that the Lys-887 substitute is able to interact spontaneously with both EC33 and GluPO_3_H_2_ (Fig 4B). For the EC33 inhibitor, the calculated energies were -59.64 ± 5.05 kcal/mol for the Arg-887 of the wild-type hAPA, -6.58 ± 1.35 kcal/mol for the Ala-887 of the R887A mutant and -27.19 ± 6.35 kcal/mol for the Lys-887 of the R887K mutant. For the GluPO_3_H_2_ inhibitor, the calculated values were -59.26 ± 9.32 kcal/mol for the Arg-887 of the wild-type hAPA, -11.15 ± 1.03 kcal/mol for the Ala-887 of the R887A mutant, and -20,22 ± 3.58 kcal/mol for the Lys-887 of the R887K mutant. Furthermore, the distance between the ε nitrogen atom from the Lys-887 sidechain of the R887K mutant and the sulfonate moiety of the EC33 inhibitor fluctuated from 3.06 to 6.39 Å during the entire simulation, suggesting that this substitution was not engaged in a stable conformation with the EC33 inhibitor (S2 Fig), which might explain why this mutant was still impacted regarding inhibitor binding. Together those results imply that, despite still being able to interact with the EC33 inhibitor, Lys-887 is not as efficient as Arg-887 from the wild-type APA. We then analyzed the effect of the mutations on the volume of the S1 subsite. For this purpose, we extracted the binding site volume information from our MD simulations trajectories (Fig 4C, S1, S2 and S3 Movies). Although the lysine being a bulkier amino acid than alanine, the binding site volume of this mutant was significantly higher than that of R887A or wild-type hAPA, regardless of the inhibitor (EC33 or GluPO_3_H_2_). The binding pocket volume averages for the APA + EC33 simulation were, 1194 ± 196 Å^3^, 821 ± 130 Å^3^ and 1828 ± 253 Å^3^ for wild-type, R887A and R887K, respectively. For the hAPA + GluPO_3_H_2_ system, those values were 831 ± 137 Å^3^, 833 ± 149 Å^3^ and 1634 ± 206 Å^3^ for wild-type, R887A and R887K, respectively. The change in the volume of the S1 subsite observed with the R878K mutant, may impair the binding and/or the optimal positioning of the substrate as well as its hydrolysis, resulting in a lower cleavage efficiency (*k*_*cat*_*/K*_*m*_) of GluNA and a lower inhibitory potency of GluPO_3_H_2_ with the R878K mutated enzyme when compared to the wild-type enzyme. This strengthened the conclusion that Arg-878 is located in the mAPA S1 subsite and therefore may drive APA substrate specificity for N-terminal acidic amino acid residues. These changes were more marked in the absence than in the presence of Ca^2+^, supporting that Arg-878 together with Ca^2+^ plays a crucial role in APA acidic substrate specificity.

### Hydrolysis of various substrates by wild-type and mutated His-mAPAs

**Fig 5:**
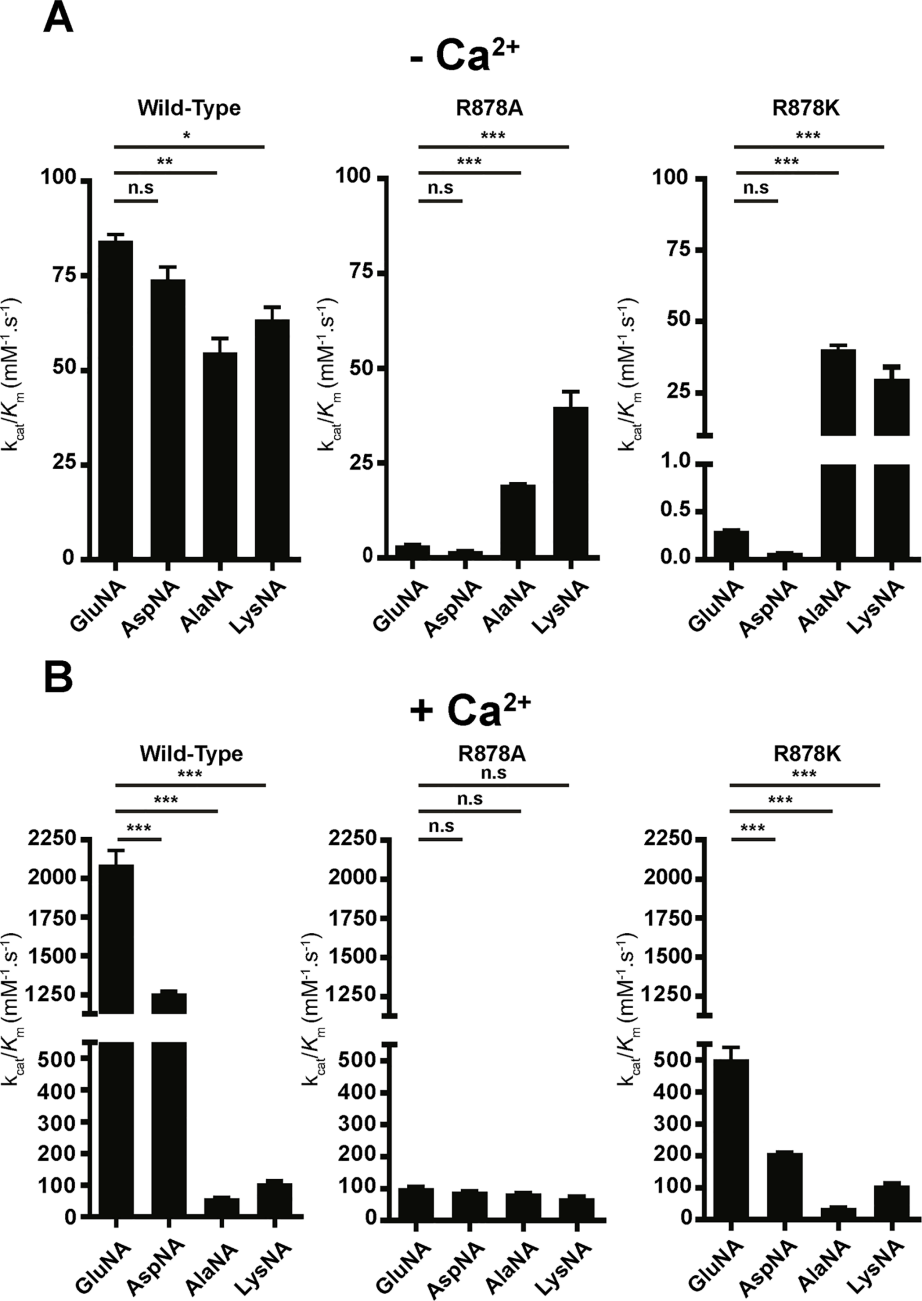
Hydrolysis of various substrates, GluNA, AspNA, AlaNA and LysNA, by recombinant mAPAs. Recombinant wild-type and mutated mAPAs hydrolysis efficiencies were evaluated using GluNA, AspNA, AlaNA and LysNA as a substrate in the absence (A) or presence (B) of 4 mM Ca^2+^. The data are the mean ± S.E of five to seven experiments performed in duplicate. *** *p*< 0,001; ** *p*< 0,01; * *p*< 0,05 and n.s (not significant) when compared with the value obtained for GluNA.

To strengthen the hypothesis that Arg-878 is involved in substrate specificity for N-terminal acidic amino acid residues, we evaluated the efficiency of hydrolysis of the wild-type and mutated His-mAPAs with various substrates differing by their side-chains. We therefore determined the kinetic parameters of wild-type and the mutated enzymes for the substrates, GluNA, AspNA, AlaNA and LysNA, in the absence or presence of Ca^2+^ and we compared their substrate hydrolysis profiles (Fig 5, S1 and S2 Tables). In the absence of Ca^2+^, the substrate hydrolysis profile for wild-type mAPA was GluNA = AspNA > LysNA > AlaNA. This profile was changed by Arg-878 substitutions. The substrate profile for R878A was LysNA > AlaNA > GluNA = AspNA and for R878K AlaNA = LysNA >> GluNA > AspNA. The analysis of the kinetics parameters of wild-type and mutated mAPAs showed that the modifications in the substrate hydrolysis profile observed for the mutated mAPAs were due to an increased substrate hydrolysis velocity and to a better affinity of the mutated enzymes for AlaNA and LysNA when compared to that for GluNA or AspNA.

The addition of Ca^2+^ amplifies the preference of wild-type mAPA for acidic substrates. Thus, GluNA and AspNA were much more efficiently hydrolyzed than LysNA, which was hydrolyzed more efficiently than AlaNA (GluNA > AspNA >> LysNA > AlaNA). This profile was partially maintained in R878K with the conservative mutation. In contrast, the R878A mutant in the presence of Ca^2+^, similarly hydrolyzed GluNA, AspNA, AlaNA and LysNA. Altogether, these data confirmed that Arg-878 participates with Ca^2+^ to APA acidic substrate specificity. Additionally, in the presence of Ca^2+^, the hydrolysis efficiency of GluNA by R878K and R878A remained 4- and 21-fold weaker than that of the wild-type, respectively. Since in the cerebrospinal fluid, the Ca^2+^ concentration fluctuates from 4.3 mM to 5.9 mM [36], which is 40 times higher than the EC_50_ of Ca^2+^ for APA. This suggests the constant presence of Ca^2+^ in the S1 subsite of endogenous APA, which consequently recognizes only APA substrates or inhibitors with an N-terminal acidic side chain, highlighting the key role of Arg-878 with Ca^2+^ in APA substrate specificity.

In conclusion, we showed that the guanidium moiety of Arg-878 in the mAPA S1 subsite interacts with the N-terminal acidic amino acid residue of the substrate or inhibitor, participating with Ca ^2+^ to the substrate specificity of APA for N-terminal acidic amino acid residues. We also showed that Arg-878 contributes to ensure an optimal positioning of the substrate during catalysis, thereby optimizing the hydrolysis of the substrate scissile peptide bond. In addition, our work showed that, whereas Arg-878 is far from the consensus sequence HEXXH and other active site contributing residues in term of primary sequence, this residue is very close from the active site with regard to the 3D structure. Indeed, the X-ray structure of APA shows that the C-terminal domain that contains the Arg-878 residue is in close contact to the region of the active site and complement by this way the structure of the S1 subsite. These new insights into the organization of the APA S1 subsite showed that this subsite is at the interface of the N- and C-terminal domains of APA and that the C-terminal domain has not only a chaperone activity as previously demonstrated but also a functional role in the S1 subsite, in substrate recognition [22, 37].

## Supporting information

S1 Table Kinetic parameters for wild type and mutated mAPAs in absence of calcium, using different synthetic substrates. *K*_*m*_ and *k*_*cat*_ values are the mean ± S.E.M from six to eight separate experiments performed in duplicate.
(DOCX)

S2 Table Kinetic parameters for wild type and mutated mAPAs in presence of calcium, using different synthetic substrates. *K*_*m*_ and *k*_*cat*_ values are the mean ± S.E.M from six to eight separate experiments performed in duplicate.
(DOCX)

S3 Table List of docked molecules used to identify key residues of the APA S1 subsite. (DOCX)

S4 Table X-Ray based score penalties applied during molecular docking. (DOCX)

S1 Fig Sequence alignment of hAPA and mAPA. Conserved residues are shaded in black, semi-conserved residues are shaded in grey. The secondary structure information was extracted from the 4KXD pdb structure, where red, blue and yellow indicate α-helix, 3_10_-helix, and extended structure (β-sheets), respectively. Active site residues are highlighted by an orange box. Residues responsible for Zn^2+^ binding are marked by orange diamonds, the catalytic glutamate is marked by a green triangle, and the glutamate responsible for N-terminus recognition is marked by a blue triangle. The mouse Arg-878 and the homologous residue in the human sequence Arg-887 are shaded in red and marked by an arrow.
(TIF)

S2 Fig Evolution of the distance between the epsilon amide from Lys-887 of the R887K mutant and the sulfonate moiety of the EC33 inhibitor. Snapshot ‘A’ corresponds to the one where the lowest distance was observed. Snapshot ‘B’ corresponds to a conformation where the distances were the same as the average of all frames. Snapshot ‘C’ corresponds to the largest distance observed. In A, B and C, the inhibitor carbon atoms are colored in orange, Zn^2+^ is colored in ochre, Ca^2+^ ion is colored in purple. The Lys-887 residue is depicted as translucent surface representation over a licorice representation in order to display bond distances with the sulfonate moiety of the EC33 inhibitor.
(TIF)

S1 Movie Analysis of the binding site volume through the MD simulation trajectory (50 ns) of the wild-type humanAPA. (MPG)

S2 Movie Analysis of the binding site volume through the MD simulation trajectory (50 ns) of the R887A humanAPA. (MPG)

S3 Movie Analysis of the binding site volume through the MD simulation trajectory (50 ns) of the R887K humanAPA. (MPG)
