# Supplementary material for: Involvement of arginine 878 together with Ca2+ in mouse aminopeptidase A substrate specificity for N-terminal acidic amino-acid residues
Source: PLoS One. 2017 Sep 6;12(9):e0184237. doi: 10.1371/journal.pone.0184237 (PMC5587309; doi:10.1371/journal.pone.0184237)
Supplement: S1 Table — Km and kcat values are the mean ± S.E.M from six to eight separate experiments performed in duplicate. (DOCX) [file pone.0184237.s001.docx]

**S1 Table. Kinetic parameters for wild type and mutated mAPAs in absence of calcium using different synthetic substrates**

*K_m_* and *k_cat_* values are the mean ± S.E.M from six to eight separate experiments performed in duplicate

| **Substrate** | **WT** | | |  | **R878A** | | |  | **R878K** | | |
| --- | --- | --- | --- | --- | --- | --- | --- | --- | --- | --- | --- |
|  | ***K_m_***  **(µM)** | ***k_cat_***  **(s^-1^)** | ***k_cat_*/*K_m_***  **(s^-1^/mM)** |  | ***K_m_***  **(µM)** | ***k_cat_***  **(s^-1^)** | ***k_cat_*/*K_m_***  **(s^-1^/mM)** |  | ***K_m_***  **(µM)** | ***k_cat_***  **(s^-1^)** | ***k_cat_*/*K_m_***  **(s^-1^/mM)** |
| **HGluβNA** | 165 ± 13.5 | 13.9 ± 0.1 | 84.3 ± 4.6 |  | 1500 ± 79.6*** | 4.95 ± 0.1*** | 3.3 ± 0.8*** |  | 12500 ± 1450*** | 3.12 ± 0.4*** | 0.3 ± 0.1*** |
| **HAspβNA** | 149 ± 15.6 | 10.4 ± 0.6 | 74.1 ± 9.2 |  | 1220 ± 24.9*** | 1.89 ± 0.2*** | 1.8 ± 0.2*** |  | 3150 ± 328*** | 0.2 ± 0.01*** | 0.07 ± 0.006*** |
| **HAlaβNA** | 394 ± 20 | 21.6 ± 0.5 | 54.9 ± 5.0 |  | 651 ± 43.8*** | 12.6 ± 0.04*** | 19.4± 0.5** |  | 670 ± 25.2*** | 27.1 ± 4.8** | 40.3 ± 4.0** |
| **HLysβNA** | 304 ± 49.7 | 16.4 ± 0.7 | 63.5 ± 8.7 |  | 394 ± 57.0** | 13.8 ± 0.7*** | 39.9 ± 5.0** |  | 336 ± 54.3^n.s^ | 9.0 ± 0.5*** | 30.1 ± 3.7** |

n.s, non significant and ***p*<0.01; *** *p*<0.001, significant when compared to the corresponding wild type value
