# Supplementary material for: Involvement of arginine 878 together with Ca2+ in mouse aminopeptidase A substrate specificity for N-terminal acidic amino-acid residues
Source: PLoS One. 2017 Sep 6;12(9):e0184237. doi: 10.1371/journal.pone.0184237 (PMC5587309; doi:10.1371/journal.pone.0184237)
Supplement: S2 Table — Km and kcat values are the mean ± S.E.M from six to eight separate experiments performed in duplicate. (DOCX) [file pone.0184237.s002.docx]

**S2 Table. Kinetic parameters for wild type and mutated mAPAs in presence of calcium using different synthetic substrates**

*K_m_* and *k_cat_* values are the mean ± S.E.M from six to eight separate experiments performed in duplicate

| **Substrate** | **WT** | | |  | **R878A** | | |  | **R878K** | | |
| --- | --- | --- | --- | --- | --- | --- | --- | --- | --- | --- | --- |
|  | ***K_m_***  **(µM)** | ***k_cat_***  **(s^-1^)** | ***k_cat_*/*K_m_***  **(s^-1^/mM)** |  | ***K_m_***  **(µM)** | ***k_cat_***  **(s^-1^)** | ***k_cat_*/*K_m_***  **(s^-1^/mM)** |  | ***K_m_***  **(µM)** | ***k_cat_***  **(s^-1^)** | ***k_cat_*/*K_m_***  **(s^-1^/mM)** |
| **HGluβNA** | 39.1 ± 3.9 | 81.7 ± 4.0 | 2090 ± 252 |  | 221 ± 4.0*** | 22.4 ± 0.1*** | 102 ± 16*** |  | 269 ± 5.1*** | 135 ± 1.3*** | 502 ± 11*** |
| **HAspβNA** | 54.0 ± 1.4 | 68.1 ± 1.3 | 1260 ± 35 |  | 199 ± 10.1*** | 17.9 ± 0.2*** | 91 ± 5*** |  | 202 ± 5.7*** | 42.2 ± 1.1*** | 209 ± 8*** |
| **HAlaβNA** | 321 ± 41 | 17.4 ± 0.6 | 59 ± 5 |  | 544 ± 74*** | 41.5 ± 1.6*** | 84 ± 9** |  | 821 ± 142*** | 26.2 ± 1.5*** | 38 ± 5** |
| **HLysβNA** | 244 ± 29.5 | 25.8 ± 0.2 | 106 ± 2.3 |  | 159 ± 7.8*** | 11.6 ± 0.2*** | 70.1 ± 9.9** |  | 229 ± 16.2^n.s^ | 24.4 ± 0.5^n.s^ | 107 ± 16** |

n.s, non significant and ***p*<0.01; *** *p*<0.001, significant when compared to the corresponding wild type value
