# Supplementary material for: Involvement of arginine 878 together with Ca2+ in mouse aminopeptidase A substrate specificity for N-terminal acidic amino-acid residues
Source: PLoS One. 2017 Sep 6;12(9):e0184237. doi: 10.1371/journal.pone.0184237 (PMC5587309; doi:10.1371/journal.pone.0184237)
Supplement: S3 Table — (DOCX) [file pone.0184237.s003.docx]

**S3 Table.** **List of docked molecules used to identify key residues of the APA S1 subsite**

| **Internal name** | **IUPAC name** | **Molecular formula** | **Reference** |
| --- | --- | --- | --- |
| EC33 | (3S)‐3‐amino‐4‐sulfanylbutane‐1‐sulfonic acid | C_4_H_11_NO_3_S_2_ | (1) |
| GluPO_3_H_2_ | (4R)-4-amino-4-phosphonobutanoic acid | C_4_H_10_NO_5_P | (2) |
| GluSH | (4S)-4-amino-5-sulfanylpentanoic acid | C_5_H_11_NO_2_S | (3) |
| MetSH | (2S)-2-amino-5-(methylsulfanyl)butane-1-thiol | C_5_H_13_NS_2_ | (4) |
| LysSH | (2S)-2,6-diaminohexane-1-thiol | C_6_H_16_N_2_S | (5) |

1. Chauvel, E. N., Coric, P., Llorens-Cortes, C., Wilk, S., Roques, B. P., and Fournie-Zaluski, M. C. (1994) Investigation of the active site of aminopeptidase A using a series of new thiol-containing inhibitors. *Journal of medicinal chemistry* **37**, 1339-1346

2. Lejczak, B., De Choszczak, M. P., and Kafarski, P. (1993) Inhibition of aminopeptidases by phosphonic acid and phosphinic acid analogues of aspartic and glutamic acids. *Journal of enzyme inhibition* **7**, 97-103

3. Wilk, S., and Thurston, L. S. (1990) Inhibition of angiotensin III formation by thiol derivatives of acidic amino acids. *Neuropeptides* **16**, 163-168

4. Reaux, A., de Mota, N., Zini, S., Cadel, S., Fournie-Zaluski, M. C., Roques, B. P., Corvol, P., and Llorens-Cortes, C. (1999) PC18, a specific aminopeptidase N inhibitor, induces vasopressin release by increasing the half-life of brain angiotensin III. *Neuroendocrinology* **69**, 370-376

5. Iturrioz, X., Vazeux, G., Celerier, J., Corvol, P., and Llorens-Cortes, C. (2000) Histidine 450 plays a critical role in catalysis and, with Ca2+, contributes to the substrate specificity of aminopeptidase A. *Biochemistry* **39**, 3061-3068
