# Supplementary material for: Involvement of arginine 878 together with Ca2+ in mouse aminopeptidase A substrate specificity for N-terminal acidic amino-acid residues
Source: PLoS One. 2017 Sep 6;12(9):e0184237. doi: 10.1371/journal.pone.0184237 (PMC5587309; doi:10.1371/journal.pone.0184237)
Supplement: S4 Table — (DOCX) [file pone.0184237.s004.docx]

**S4 Table. X-Ray based score penalties applied during molecular docking**

| **Score penalty** | **Involved residues** | **Description** |
| --- | --- | --- |
| 20 | Glu-215, Glu-352, Glu-386, Glu-408 | Bind to amino-termini of ligands; Crystallographic structures contains 2-4 bonds in this cluster. |
| 10 | Arg-878 | Close to bound Ca^2+^ in crystallographic structures; should be targeted by any acid group bound near the Ca^2+^. |
| 5 | Tyr-471 | Secondary interaction performed by all X-ray references. |
