# Supplementary figures and images for: Involvement of arginine 878 together with Ca2+ in mouse aminopeptidase A substrate specificity for N-terminal acidic amino-acid residues
Source: PLoS One. 2017 Sep 6;12(9):e0184237. doi: 10.1371/journal.pone.0184237 (PMC5587309; doi:10.1371/journal.pone.0184237)

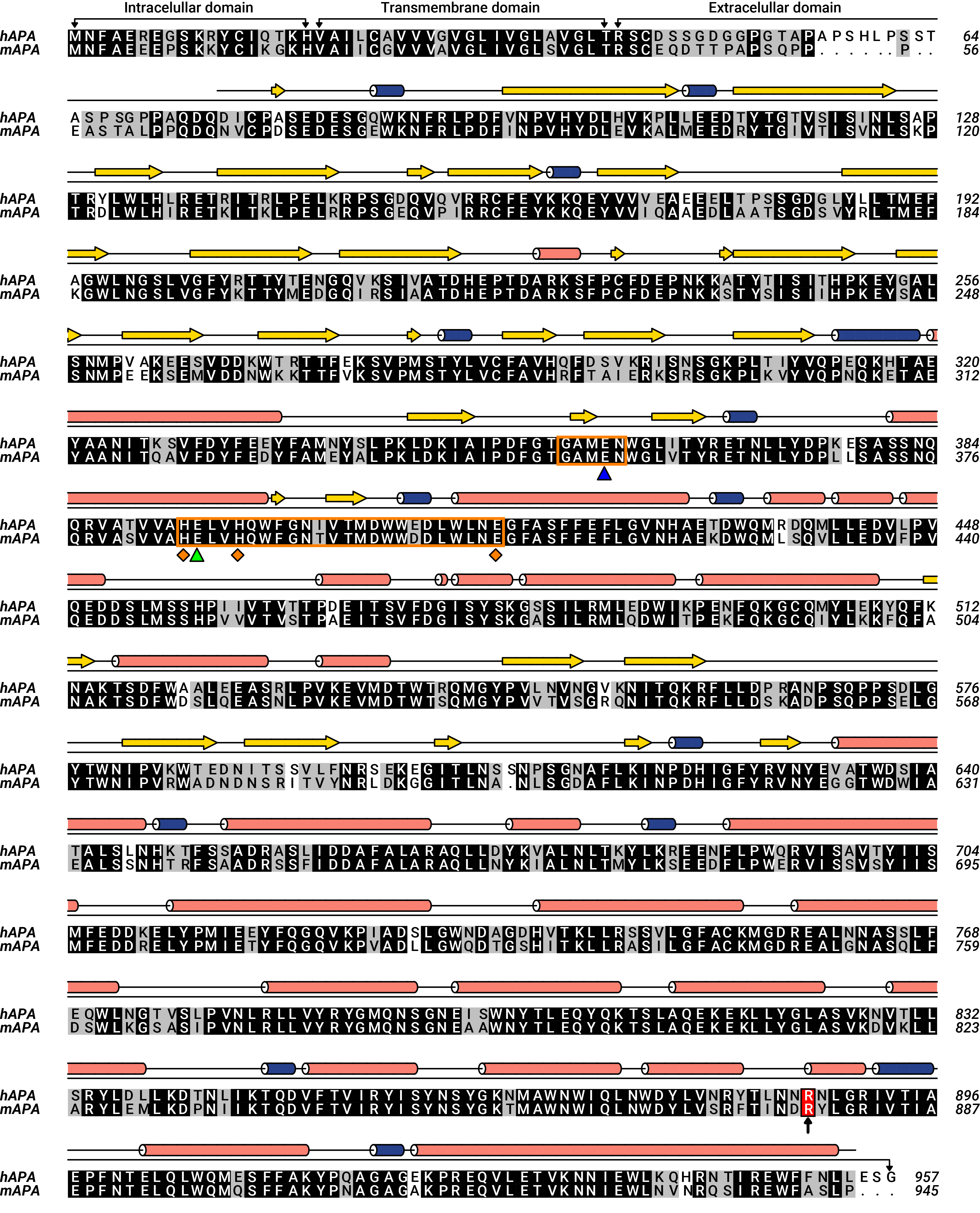

Supplement: S1 Fig — Conserved residues are shaded in black, semi-conserved residues are shaded in grey. The secondary structure information was extracted from the 4KXD pdb structure, where red, blue and yellow indicate α-helix, 310-helix, and extended structure (β-sheets), respectively. Active site residues are highlighted by an orange box. Residues responsible for Zn2+ binding are marked by orange diamonds, the catalytic glutamate is marked by a green triangle, and the glutamate responsible for N-terminus recognition is marked by a blue triangle. The mouse Arg-878 and the homologous residue in the human sequence Arg-887 are shaded in red and marked by an arrow. (TIF) [file pone.0184237.s005.tif]

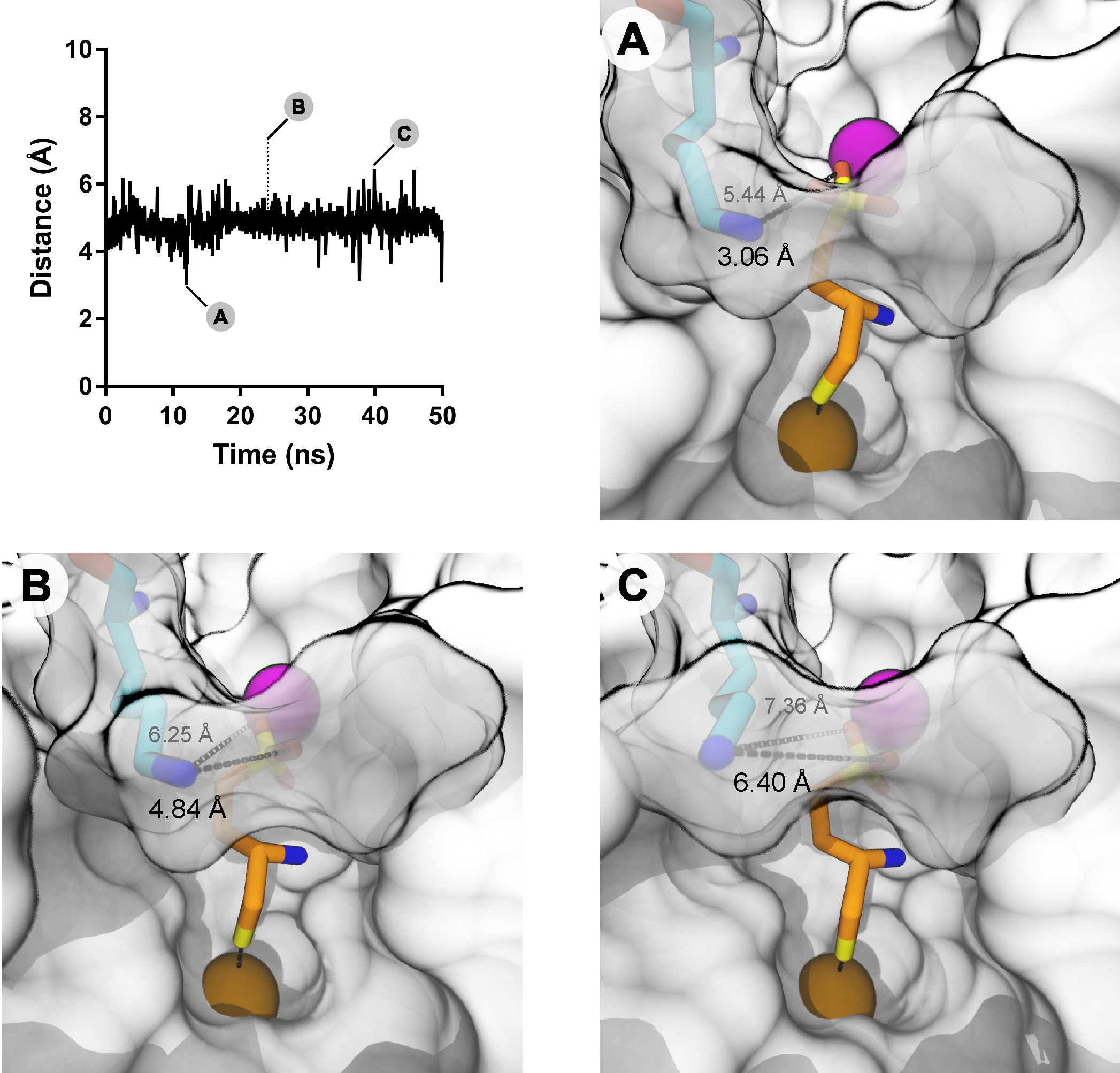

Supplement: S2 Fig — Snapshot ‘A’ corresponds to the one where the lowest distance was observed. Snapshot ‘B’ corresponds to a conformation where the distances were the same as the average of all frames. Snapshot ‘C’ corresponds to the largest distance observed. In A, B and C, the inhibitor carbon atoms are colored in orange, Zn2+ is colored in ochre, Ca2+ ion is colored in purple. The Lys-887 residue is depicted as translucent surface representation over a licorice representation in order to display bond distances with the sulfonate moiety of the EC33 inhibitor. (TIF) [file pone.0184237.s006.tif]
